# Current ART, determinants for virologic failure and implications for HIV drug resistance: an umbrella review

**DOI:** 10.1186/s12981-023-00572-6

**Published:** 2023-10-27

**Authors:** SeyedAhmad SeyedAlinaghi, Amir Masoud Afsahi, Ali Moradi, Zohal Parmoon, Pedram Habibi, Pegah Mirzapour, Mohsen Dashti, Afsaneh Ghasemzadeh, Elaheh Karimi, Foziye Sanaati, Zahra Hamedi, Ayoob Molla, Esmaeil Mehraeen, Omid Dadras

**Affiliations:** 1 Iranian Research Center for HIV/AIDS, Iranian Institute for Reduction of High-Risk Behaviors, Tehran University of Medical Sciences, Tehran, Iran; 2 Department of Radiology, School of Medicine, University of California, San Diego (UCSD), San Diego, CA USA; 3 School of Medicine, Tehran University of Medical Sciences, Tehran, Iran; 4 Department of Radiology, Tabriz University of Medical Sciences, Tabriz, Iran; 5 School of Nursing and Allied Medical Sciences, Maragheh University of Medical Sciences, Maragheh, Iran; 6 Shahid Beheshti University of Medical Sciences, Tehran, Iran; 7 School of Medicine, Bushehr University of Medical Sciences, Bushehr, Iran; 8 Department of Health Information Technology, Khalkhal University of Medical Sciences, Khalkhal, 5681761351 Iran; 9 Bergen Addiction Research, Department of Addiction Medicine, Haukland University Hospital, Bergen, Norway

**Keywords:** Antiretroviral therapy, ART, Virologic failure, HIV, AIDS

## Abstract

**Objective:**

The purpose of this study is to investigate the incidence of determinants for virologic failure and to identify predisposing factors to enhance treatment efficacy. Tackling this global public health issue is the key to reducing the rate of virological failure and increasing the success of treatment for those living with HIV.

**Methods:**

This umbrella review delves into various aspects of current anti-retroviral therapy (ART) which is the primary treatment for human immunodeficiency virus (HIV) infection. Comprehensive searches were conducted in online databases including PubMed, Embase, Scopus, and Web of Science, up to May 26, 2023. Following the screening and selection of relevant articles, eligible articles were included in the data extraction. This study adhered to the PRISMA guideline to report the results and employed the NIH quality and bias risk assessment tool to ensure the quality of included studies.

**Results:**

In total, 40 review studies published from 2015 to 2023 were included. The bulk of these studies concurred on several major factors contributing to HIV drug resistance and virological failure. Key among these were medication adherence, baseline and therapeutic CD4 levels, the presence of co-infections, and the advanced clinical stage of the infection.

**Conclusion:**

The resistance to HIV drugs and instances of determinants for virologic failure have a profound impact on the life quality of those infected with HIV. Primary contributors to this scenario include insufficient adherence to treatment, decreased CD4 T-cell count, elevated viral levels, and certain treatment regimens. Implementing appropriate interventions could address these issues. Sub-Saharan Africa exhibits elevated rates of determinants for virologic failure, attributed to the delay in HIV testing and diagnosis, and late initiation of antiretroviral therapy (ART). It is essential to undertake further research aimed at enhancing the detection of resistance in HIV patients and mitigating viral failure by addressing these underlying causes.

**Supplementary Information:**

The online version contains supplementary material available at 10.1186/s12981-023-00572-6.

## Introduction

As of the end of 2021, HIV continues to pose a significant global challenge, affecting approximately 38.4 million individuals worldwide [1]. While there is still no cure for HIV, the early introduction of Antiretroviral Therapy (ART) has proven to be instrumental in significantly reducing mortality rates among HIV-infected individuals [2–5]. Moreover, ART has been found to reduce the viral load in genital secretions, which is closely linked to the sexual transmission of HIV to partners, potentially slowing the spread of the virus [6–8]. Consequently, initiatives such as the United Nations' 90-90-90 program have been introduced with the goal of reducing the number of new HIV cases to 500,000 per year. The program aims to diagnose 90% of people infected with HIV, ensure that 90% of diagnosed individuals receive ART, and achieve suppressed viral load in 90% of those on ART [9, 10].

Historically, the commencement of ART was delayed until CD4 counts dropped below 200, a level at which opportunistic infections may thrive [11]. However, current guidelines recommend starting ART as soon as possible after diagnosis [12, 13]. Despite the significant benefits of ART, not all individuals receiving treatment achieve viral suppression. Approximately 20% of individuals in contact with healthcare or receiving ART do not achieve viral suppression, highlighting the importance of addressing adherence challenges and virologic failure [14]. According to the World Health Organization (WHO), all individuals diagnosed with HIV should begin ART as soon as possible, and those at high risk of infection should receive pre-exposure therapy [15]. Adherence to the treatment regimen is crucial for successful treatment outcomes, as poor adherence can lead to the development of drug-resistant strains of the virus or virologic failure [16].

As new genotypes of HIV emerge, the importance of genetic diversity is more clear in the process of treatment, vaccine design [17] and drug-resistance strains [18]. The reason for this diversity is thought to be the high rate of errors while replicating [19]. As the data suggests, a combination therapy across the available ARTs is quite effective against most HIV-1 subtypes, however, there are differences between subtypes across the globe, hence, there are differences in their drug resistance [20]. To mention an example, subtype C can go through K65R mutation, related to tenofovir resistance, more than subtype B [21, 22]. HIV drug resistance is a genuine challenge in some countries trying to adhere to the goal of UNAIDS’ 90/90/90 goals [10].

Several factors influence adherence to ART and virologic outcomes in HIV-positive individuals. Patient-related factors, such as substance and alcohol abuse, unstable housing, financial difficulties, and psychiatric disorders, can all contribute to non-adherence [23, 24]. Additionally, the complexity of the treatment regimen, including the number of pills and side effects, can also impact adherence [25]. Thus, it is essential to address these determinants to improve treatment success and reduce virological failure among HIV patients on ART. Measuring virologic failure is vital in monitoring treatment effectiveness. Detectable viremia, measured by PCR, may not always indicate clinical significance, but ART is still recommended even when viremia levels are below 200 [26–29]. Factors such as poor adherence to the therapeutic regimen, gastrointestinal malabsorption, or drug interactions can contribute to detectable viremia [26–29]. By understanding and addressing the determinants of virologic failure, we can enhance the effectiveness of antiretroviral therapy and contribute to the global effort in tackling this public health issue. Therefore, this study aims to investigate the status of determinants for virologic failure and also predisposing factors by reviewing the current literature.

## Methods

This comprehensive review investigates currently prescribed antiretroviral agents in the course of human immunodeficiency virus (HIV) infection. The primary focus is to explore evidence of determinants for virologic failure and its implications for drug resistance to antiretroviral therapy (ART) agents. To ensure transparent reporting, this review adheres to the guidelines of the Preferred Reporting Items for Systematic Reviews and Meta-Analyses (PRISMA). Additionally, the methodological integrity of the included publications is assessed using the National Institutes of Health (NIH) quality and bias risk assessment tool.

### Data sources

We conducted a systematic search on four major databases, namely PubMed/MEDLINE, Embase, Scopus, and Web of Science (WoS), up to May 26, 2023. To maximize the identification of relevant publications, we employed Boolean operators between the keywords and performed combined keyword searches. The search inquiry of PubMed/MEDLINE is as follows, with the inquiries for other databases provided as an Additional file 1.

((“HIV”[mesh] OR “Human Immunodeficiency Virus”[Title/Abstract] OR “HIV”[Title/Abstract] OR “AIDS Virus*”[Title/Abstract] OR “Acquired Immune Deficiency Syndrome Virus”[Title/Abstract] OR “Acquired Immunodeficiency Syndrome Virus”[Title/Abstract] OR “aids associated virus”[Title/Abstract] OR “aids related virus”[Title/Abstract] OR “immunodeficiency associated virus”[Title/Abstract]) AND (“Antiretroviral Therapy, Highly Active”[mesh] OR “Antiretroviral Therapy, Highly Active”[Title/Abstract] OR “anti-retroviral therapy”[Title/Abstract] OR “ART (drug therapy)”[Title/Abstract] OR “antiretroviral therapy”[Title/Abstract] OR “HAART”[Title/Abstract]) AND (“Drug Resistance, Viral”[mesh] OR “Drug Resistance, Viral”[Title/Abstract] OR “Antiviral Drug Resistance”[Title/Abstract] OR “Drug Resistance”[Title/Abstract] OR “antiviral resistance”[Title/Abstract] OR “virologic failure”[Title/Abstract]).

As the searching of databases was finished and the relevant articles were obtained, we gathered them in a single file of EndNote to remove duplicates and further to steps of selection and data extraction.

### Study selection

A two-prong approach was used for article screening and selection. In the first step, two researchers evaluated the relevance of articles based on their titles and abstracts. In the second step, three other researchers reviewed the full texts of the screened literature. Publications that met the following criteria were included for data extraction:

Inclusion criteria

- Review articles addressing ART in HIV infection, virologic failure of ART, and resistance to ART in HIV-positive patients.
- Articles written in English and published in peer-reviewed journals.

Exclusion criteria

- Case series, and case reports, editorial letters, opinion letters, anecdotal records, and records published in non-academic sources like magazines, websites, and social media.
- Non-human research studies, ongoing trial studies without published data, and abstracts without full texts.

### Data extraction

Articles deemed relevant to the study objectives after the second step of selection and met the eligibility criteria were included for data extraction. Details of the extracted data are presented in Table 1. Three researchers were involved in this process, and the accuracy of the extracted data was double-checked by other team members to eliminate duplicates and address the inconsistencies in the reported results.

**Table 1:** Bias risk assessment of the studies by NIH QA Tool for systematic reviews and meta-analysis

First Autor (# of Ref)	*Question								Rating by reviewers	
	1	2	3	4	5	6	7	8	# Reviewer1	# Reviewer2
Aberg, J. A. [30]	Yes	Yes	NA	NA	NA	Yes	NA	NA	Poor**	Poor**
Agegnehu, C. D. [31]	Yes	Yes	Yes	Yes	Yes	Yes	Yes	Yes	Good	Good
Almeida-Brasil, C. C. [32]	Yes	Yes	Yes	Yes	Yes	Yes	Yes	Yes	Good	Good
Anderson, K. [33]	Yes	Yes	Yes	NA	NA	Yes	NA	NA	Fair	Fair
Bernabé, K. J. [34]	Yes	Yes	Yes	Yes	No	Yes	No	No	Good	Good
Bertagnolio, S.[35]	Yes	Yes	Yes	Yes	Yes	Yes	Yes	Yes	Good	Good
Bezabhe, W. M. [36]	Yes	Yes	Yes	Yes	Yes	Yes	Yes	Yes	Good	Good
Blanco, J. L. [37]	Yes	Yes	NA	NA	NA	Yes	NA	NA	Poor**	Poor**
Borges Á, H. [38]	Yes	Yes	Yes	Yes	Yes	Yes	Yes	Yes	Good	Good
Cevik, M. [39]	Yes	Yes	NA	NA	NA	Yes	NA	NA	Poor**	Poor**
Cruciani, M. [40]	Yes	Yes	Yes	Yes	Yes	Yes	Yes	Yes	Good	Good
Cruz, M. L. [41]	Yes	Yes	Yes	NA	NA	Yes	NA	NA	Fair	Fair
Daltro, A. C. B. [42]	Yes	Yes	Yes	Yes	CD	Yes	No	NA	Fair	Fair
de Waal, R. [43]	Yes	Yes	NA	NA	NA	NA	NA	NA	Poor**	Poor**
Diallo, M. [44]	Yes	Yes	Yes	Yes	CD	Yes	No	NA	Fair	Fair
Edessa, D. [45]	Yes	Yes	Yes	Yes	Yes	Yes	Yes	Yes	Good	Good
Feng, Q. [46]	Yes	Yes	Yes	Yes	Yes	Yes	Yes	Yes	Good	Good
Grau, S. [47]	Yes	Yes	Yes	No	Yes	No	NA	NA	Fair	Fair
Guo, C. [48]	Yes	Yes	Yes	Yes	Yes	Yes	Yes	Yes	Good	Good
Gupta, R. K. [49]	Yes	Yes	Yes	Yes	Yes	Yes	Yes	Yes	Good	Good
Hauser, A. [50]	Yes	Yes	Yes	Yes	Yes	Yes	Yes	Yes	Good	Good
Huang, Y. [51]	Yes	Yes	Yes	Yes	Yes	Yes	Yes	Yes	Good	Good
Kabbara, W. K. [52]	Yes	NA	Yes	NA	NA	Yes	NA	NA	Poor**	Poor**
Kandel, C. E. [53]	Yes	NA	NA	NA	NA	NA	NA	NA	Poor**	Poor**
Karade, S. [54]	Yes	Yes	Yes	Yes	Yes	Yes	NA	NA	Fair	Fair
Kefale, A. T. [55]	Yes	Yes	Yes	Yes	Yes	Yes	Yes	Yes	Good	Good
Llibre, J. M. [56]	Yes	Yes	Yes	Yes	Yes	Yes	NA	NA	Fair	Fair
Mbuagbaw, L. [57]	Yes	Yes	Yes	Yes	Yes	Yes	Yes	Yes	Good	Good
Mbunkah, H. A. [58]	Yes	Yes	Yes	NA	Yes	Yes	NA	NA	Fair	Fair
McCluskey, S. M. [59]	Yes	NA	NA	NA	NA	NA	NA	NA	Poor**	Poor**
Muheem, A. [60]	Yes	Yes	Yes	CD	No	Yes	NA	NA	Fair	Fair
Nozza, S. [61]	Yes	NA	NA	NA	NA	Yes	NA	NA	Poor**	Poor**
Patel, R. [62]	Yes	Yes	Yes	Yes	No	Yes	NA	NA	Fair	Fair
Ssemwanga, D. [63]	Yes	CD	Yes	Yes	NA	Yes	NA	NA	Fair	Fair
Stella-Ascariz, N. [64]	Yes	CD	Yes	Yes	NA	Yes	NA	NA	Fair	Fair
Temereanca, A. [65]	Yes	NA	NA	Yes	NA	NA	NA	NA	Poor**	Poor**
Vannappagari, V. [66]	Yes	Yes	Yes	Yes	Yes	Yes	NA	NA	Fair	Fair
Wallis, C. L. [67]	Yes	NA	NA	NA	NA	NA	NA	NA	Poor**	Poor**
Xing, H. [68]	Yes	NA	NA	NA	NA	NA	NA	NA	Poor**	Poor**
Zuo, L. [69]	Yes	Yes	Yes	Yes	Yes	Yes	Yes	Yes	Good	Good

*NIH* National Institutes of Health, *CD* cannot determine, *NR* not reported, *NA* not applicable

^*^ The NIH Quality Assessment Tool for Systematic Reviews and Meta-Analyses (https://www.nhlbi.nih.gov/health-topics/study-quality-assessment-tools) contains eight questions: 1 Is the review based on a focused question that is adequately formulated and described? 2 Were eligibility criteria for included and excluded studies predefined and specified? 3 Did the literature search strategy use a comprehensive, systematic approach? 4 Were titles, abstracts, and full-text articles dually and independently reviewed for inclusion and exclusion to minimize bias? 5 Was the quality of each included study rated independently by two or more reviewers using a standard method to appraise its internal validity? 6 Were the included studies listed along with important characteristics and results of each study? 7 Was publication bias assessed? 8 Was heterogeneity assessed? (This question applies only to meta-analyses.)

^**^ Due to the type of these studies, which are literature review, assessing some questions were not applicable

### Bias risk assessment

To evaluate the bias risk of the included studies, we utilized the National Institute of Health (NIH) quality assessment (QA) tool. Two independent researchers completed the assessment. Table 1 displays the results of their assessment for each study. The 8 items of this tool are depicted underneath the table.

## Results

**Fig. 1:**
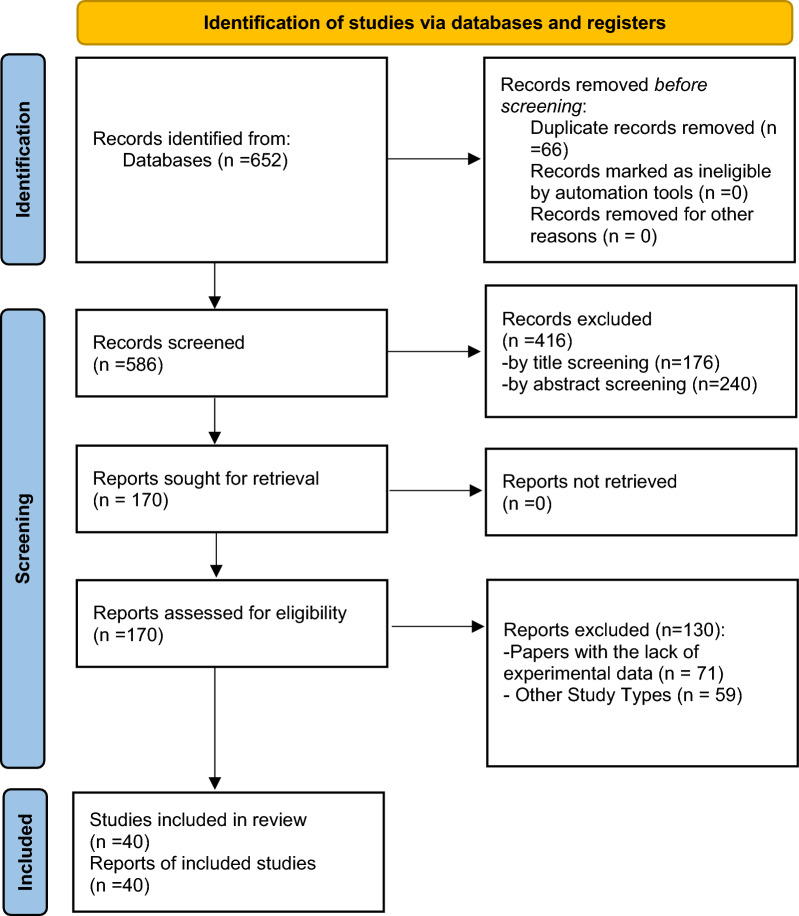
PRISMA 2020 flow diagram of study retrieval process

The initial search strategy yielded a total of 652 papers. Following the first review of papers, 66 duplicates were removed, and two researchers independently assessed the titles and abstracts of the remaining 586 articles. Next, the remaining articles were subjected to a full-text screening where eligibility criteria were applied; 40 articles finally fulfilled the inclusion criteria and were included in this umbrella review (Fig. 1). The quality assessment of included studies was performed using the NIH quality assessment tool and is presented in Table 1.

Of 40 eligible articles, 16 were review articles, 17 were meta-analyses with quantitative synthesis, and the remaining 7 were systematic reviews. More than 1250 studies were enrolled in our review; involving more than 427,058 patients with HIV infection. The number of included studies and the study population in ten and twenty reviews, respectively, were not clearly stated. The included studies were published between 2015 and 2023, covering a broad time frame.

Three studies were specifically conducted among adolescents with perinatally acquired HIV infection, while the remaining 37 involved adults. The umbrella review's primary focus was on the current understanding of determinants for virologic failure risks and implications for drug resistance in ongoing antiretroviral therapy (ART) strategies. Thus, the review examined and listed several key items, including the first author (reference), country, year of publication, study population, included articles, type of ART, implications for drug resistance, virological failure risk factors, and main findings of the studies. The comprehensive analysis of these items can be found in Table 2.

**Table 2:** Description of the findings reported in eligible studies

References	Country/year	Review type	Study population	Articles included	Type of ART	Implications for drug resistance	Virologic Failure risk factors	Main findings
[30]	USA/2023	Critical review	NR	94	-PI -NNRTIs - INSTIs -Fusion inhibitors -CCR5 inhibitors -post attachment inhibitors -attachment inhibitors	A substantial risk of resistance selection may accompany treatment regimens combining long-acting injectable medications and conventional oral medications		–
[31]	Ethiopia/2022	Systematic Review and Meta‐Analysis	6638	18	NR	–	-Lower ART adherence -Longer ART duration -Lower CD4 count -Co-infection with TB	–
[32]	Brazil/2018	Systematic Review and Meta‐Analysis	18,010	38	NR	–	–	Self-report measures of adherence can predict virologic failure as well as or better than objective measures like pharmacy refills, electronic monitoring, physician assessment, and pill count. Combining different metrics did not improve the predictive value, comparatively to any single measure
[33]	South Africa/2020	literature review	N = NR (adolescents with PHIV)	34	NR	As ART was continued longer, the risk of resistance increased. In both high-income and low- and middle-income nations, there is a high occurrence of multi-drug resistance	-Higher viral load (> 1 million copies/ml) at ART initiation -lower CD4 percentage at ART initiation -lower current CD4 levels -lower CDC staging -pre-adolescent viral failure -prior exposure to PMTCT regimens -prior non-combination ART -increased number of prior ART regimens -receiving second-line regimens -use of nevirapine versus lopinavir with ritonavir -use of nevirapine versus efavirenz -use of ritonavir as a single PI	- 90% of children whose first-line ART had failed showed mutations related to resistance
[34]	South Africa/2022	Systematic Review and Meta‐Analysis	14189	36	NR	–	–	In Sub-Saharan Africa, the CD4 cell count at the time of virologic failure identification and at second-line ART switch has not significantly changed over the course of more than ten years. Besides, the time between virologic failure and second-line ART initiation is about 530 days, far longer than what is recommended by international standards
[35]	Switzerland/2020	Systematic Review and Meta‐Analysis	31441	32	NNRTIs	NNRTI-based first-line ART with PDR was associated with new resistance mutations	- NNRTI-based first-line ART with PDR	The incidence of virological failure was tripled in those with PDR, and the clinical impact was considerably greater in those with NNRTI-PDR
[36]	Australia/2016	Systematic Review and Meta‐Analysis	27905	43	-NNRTI -Boosted-PIs -Unboosted-PIs	–	−Suboptimal drug adherence	Optimal adherence to ART is related to favorable clinical outcomes, regardless of the threshold for such adherence. Adherence levels for adequate virologic outcomes appear to be lower than the conventionally accepted cut-off point (95% adherence)
[37]	Spain/ 2018	literature review	NR	8	DTG	DTG monotherapy is associated with the occurrence of drug resistance	–	DTG's robustness, defined as the absence of resistance selection following virological failure, is undisputed when combined with other active drugs in triple or even dual therapy
[38]	Denmark/2016	Systematic Review and Meta‐Analysis	9047	39	- NNRTI - PI/r	–	- NNRTI regimens specifically those based on NVP	PI/r poses a lesser probability of viral resistance emerging than NNRTI and according to analyses limited to trials with EFV as the third medication, NNRTI-based combination ART appeared to be linked with a slenderly decreased risk of discontinuation due to virologic failure
[39]	UK/2020	A Review of Published Cases	NR (INSTI-Naïve Patients on First-line or Second-line ART)	8	DTG	Emergent drug resistance can occur even in highly resistant regimens like those containing DTG due to reasons such as poor medication adherence and HIV disease factors particularly high baseline viral load and active co-infections	–	–
[40]	Italy/2019	Systematic Review and Meta‐Analysis	6407	7	DTG and non- DTG containing ART, in combination with 2 NRTIs	Prolonged exposure to RAL or EVG in the case of treatment failure, can lead to cross-resistance to DTG and BIC	–	Compared to RAL and EVG, DTG and BIC have a stronger barrier to resistance. NRTIs in combination with DTG or BIC in the initial triple-drug ART regimen did not accompany drug resistance, and only rarely has Dolutegravir resistance been observed in patients who have previously received treatment
[41]	Brazil/2015	literature review	N = NR (adolescents with PHIV)	64	NR	The difficulty in maintaining treatment compliance throughout childhood contributes to the establishment of resistance-associated mutations in the HIV virus	–	In 42% of the sexually active adolescents with PHIV, the viral load exceeded 5000 copies/mL, and practically all of these patients had mutations linked to resistance
[42]	Brazil/2023	Systematic Review	N = NR (adolescents and young adults living with HIV)	31	NR	-	-low CD4 levels -substance misuse -alcohol use -low education level -poor adherence to medications -missing doses of ART due to forgetfulness	–
[43]	South Africa/ 2018	literature review	NR	NR	NR	DTG as monotherapy is related to high risk of developing drug resistance	DTG monotherapy	Due to the high prevalence of pretreatment NNRTIs resistance mutations in sub-Saharan Africa, Dolutegravir-based first-line ART has been implemented and it appears to be particularly effective in both ART-naïve and ART-experienced patients if it is coupled with a strong NRTIs foundation
[44]	Canada/2020	Systematic Review	18985	28	- NNRTIs - NRTIs - PIs	The most common resistance mutations included the M1841/V for the NRTIs and K103N, and Y181 for the NNRTIs	-pre-ART CD4 < 200 cell/μl -low adherence	It is recommended that alternate ART regimens be taken into consideration given the rise of NRTIs and NNRTIs resistance mutations
[45]	Ethiopia/ 2019	Systematic Review and Meta‐Analysis	18550 (participants receiving second-line HIV treatment)	33	- PIs with or without ritonavir boost	–	- Poor adherence -higher baseline viral load -lower CD4 cell counts -more advanced WHO clinical stages	Failure rates were higher among children, in the southern area of sub-Saharan Africa, and between 12 and 18 months after treatment started
[46]	China/2019	Systematic Review and Meta‐Analysis	4251	12	quadruple and triple cART	Adding a fourth drug to first-line treatment might lead to lower adherence to treatment and consequently drug resistance and treatment failure	–	The effects of quadruple cART were not better than standard triple cART for first-line treatment of PWH
[47]	UK/2023	Systematic Review	369	5	- BIC/FTC/TAF - DTG/3TC - DTG/RPV	The dual ART regiments showed less drug resistance occurrence than triple therapy	–	The regimen based on dual antiviral drugs is less toxic than triple therapy and has more adherence to treatment, effectiveness in achieving or maintaining viral suppression, and as a result, it has less drug resistance than TT
[48]	China/2021	Systematic Review and Meta‐Analysis	3558	12	- NNRTIs - INIs	Prevalence of drug resistance to NNRTIs is the highest, while the drug resistance to INIs is the lowest. This may guide the selection of clinical antiretroviral drugs	–	The prevalence of drug resistance in naive people with acute HIV infection, primary HIV infection and early HIV infection is moderate and it is necessary to identify the drug resistance in developing and advanced countries before starting retroviral drugs
[49]	UK/2001–2016	Systematic review and meta-regression analysis	56044	358 datasets	- NNRTIs - NRTIs	Drug resistance in NNRTI regimen was significantly higher than NRTI regimen	–	Pre-treatment drug resistance is increasing significantly in low- and middle-income countries, specifically resistance to NNRTIs has approached the 10% WHO threshold for first-line switching
[50]	Switzerland/ 2022	Systematic Review	2690	19	- NNRTIs - NRTIs	The K65N/R gene mutation rise as a result of adding TDF to NRTI after two years, increasing drug resistance to NRTIs	–	NRTI/NNRTI drug resistance mutations are common in patients failing first-line ART in South Africa. These patients may be switched to a DTG-based regimen with compromised NRTIs, which can impair the long-term efficacy of ART
[51]	China/2018	Systematic Review and Meta‐Analysis	3773	9	LPV/r-based second-line ART	LPV/r-based regimen significantly induces viral suppression and decreases drug resistance in patients with first-line virologic failure and drug resistance	–	The use of LPV/r therapy has significant efficacy in patients who have failed first-line antiretroviral therapy
[52]	Lebanon/2015	literature review	NR	NR	-Complera (RPV/FTC/TDF)	drug resistance was higher with RPV, especially in patients with baseline viral load > 100,000 copies/mL	viral load > 100,000 copies/mL	Complera is a recommended alternative treatment in ART naïve patients who have a pre-ART plasma HIV RNA < 100,000 copies/mL and CD4 count > 200 cells/mm3
[53]	Canada/2015	literature review	NR	NR	DTG	In patients who used DTG as a first-line drug, no cases of drug resistance and virological failure were observed	–	DTG is an effective antiretroviral agent for both treatment-naïve and treatment-experienced patients infected with HIV
[54]	India/2018	Systematic Review	3353	23	- NNRTIs -NRTIs	K65R and 103N were the only NRTI and NNRTI mutations, respectively	–	Complications of resistance against drugs in the first-line regimen remained steady over the 10-year period. However, periodic monitoring is required
[55]	Ethiopia /2018	Systematic Review and Meta‐Analysis	28149	17	-TDF based-Regimens - ZDV based-Regimens	–	Virologic failure was significantly observed in patients treated with ZDV compared to those treated with TDF	Regimens based on ZDV are more effective in preventing death and suppressing viral load in infected patients. However, TDF-based regimens were better in terms of safety and tolerance
[56]	Spain/2018	Systematic Review	6175	14	- NNRTIs - NRTIs -PI -INSTIs	The increase of K65N/R gene mutation was observed as a result of drug resistance	–	Because standard definitions of virologic failure and resistance testing criteria do not exist in pivotal phase III RCTs of first-line ART, there is a possibility of underreporting of resistance mutations, particularly when genotyping is performed only at higher viral load cutoffs
[57]	Canada/2016	Systematic Review and Meta‐Analysis	3278	12	-NVP -EFV	Development of drug resistance is probably less in the EFV than NVP	–	EFV- and NVP-based regimens are both equally effective in viral suppression, preventing disease progression, and reducing mortality. EFV affects the mental performance of patients more. While NVP causes more liver damage, decreased white blood cells and rashes
[58]	Switzerland/2020	Systematic Review	1148	92	-NNRTIs -NRTIs -PIs -INSTIs	The relevance of drug resistance will depend on the prevalence of transmitted INSTI resistance, which remains low but may increase in the future; and the overall genetic barrier of INSTI regimens	–	There is a considerable range in the lower limit of detection of low-abundance drug-resistant human immunodeficiency virus-1 variants for different assays, from < 0.01% as seen with AS-PCR to 1%–5% for ultradeep sequencing assays and other methods
[59]	USA/2022	literature review	NR	2	DTG	The DTG-containing regimens have low resistance and virologic failure	-poor adherence -PDR	–
[60]	India/2021	Systematic Review	NR	46	-NNRTIs -NRTIs -PIs -INIs -fusion and CCR5 inhibitors	A combinatorial drug delivery approach using nanocarriers has the potential to reduce the emergence of drug-induced resistance	–	The combinational medication delivery method for HIV infection may provide a more effective therapeutic response compared to traditional HAART therapy and single drug-loaded nanoformulation
[61]	Italy/2015	State-of-the-art review	NR	NR	Dual therapy	–	- HIV/RNA levels > 100,000 copies/ml -CD4 levels < 200/mmc.	Dual ART is typically less effective than suggested triple regimens. As a result, this strategy should only be used cautiously in individuals who are ARV-naive and not openly recommended
[62]	UK/2021	Systematic Review	5017	73	DTG + 3TC	Dual therapy of DTG plus 3TC has long-lasting efficacy and a high barrier to resistance	–	DTG + 3TC was effective at achieving and maintaining virologic suppression in a range of people with HIV, including those who were switching from three-drug regimens to two-drug regimens
[63]	Nigeria/2015	literature review	5541	99	- NNRTIs -NRTIs -PIs	- HIV subtype diversity, patient adherence, drug stock-outs, counterfeit drugs, and lack of monitoring are specific risk factors related to drug resistance in resource-limited settings	–	HIV drug resistance prevalence in Africa is relatively high. Changing patterns of drug resistance over time, regular surveillance of ART resistance, and collecting drug resistance data for informed policies must be considered
[64]	Spain/2017	literature review	NR	NR	-NNRTIs -INSTIs -PIs	As DRMVs impact treatment efficacy, particularly for drugs with a low genetic barrier to resistance like NNRTIs, DRMVs detection through sensitive techniques is essential	NNRTI-resistant DRMVs	The impact of DRMVs on INSTIs and PIs is less certain and requires further research
[65]	Romania/2023	literature review	NR	NR	- FTR -IBA -PRO 140 - ISL -LEN	-Resistance to IBA is conferred by decreased viral expression of specific binding sites in the HIV gp120 envelope protein	–	- FTR as a long-acting CCR5 inhibitor is being considered as a life-saving option for patients with drug-resistant HIV
[66]	USA/2019	Systematic Review	> 100,000	127	- NNRTIs - NRTIs	The risk of 3TC- or RPV-resistant mutations in treatment-naive, HIV-1-infected individuals is low	–	The use of two-drug regimens such as DTG and 3TC (in treatment-naïve people) and DTG and RPV (in people who are virally suppressed in the 3-drug regimen and change to 2DR) is highly effective
[67]	South Africa/2017	literature review	614	5	NNRTI	PDR, the timing of treatment onset, HIV subtype, antiretrovirals used in first- and second-line ART, viral load monitoring, and medication adherence are associated with the emergence of drug resistance	–	–
[68]	China/2022	literature review	NR	NR	NR	-Higher CD4 T cell counts are associated with lower drug resistance and reduced viral loads	–	The mortality rate of patients developing drug-resistant HIV within 1 year after starting ART was 1.9 times higher than the mortality rate of those developing drug resistance at a later time point
[69]	China/2020	Systematic Review and Meta‐Analysis	21,451 ART-naïve and 30,475 ART-treated individuals with HIV-1 infection	170	3TC and/or EFV or NVP	As NRTI-associated mutations (M184V/I) and NNRTI-associated mutations (K103N/S, Y181C/I and G190A/S) were responsible for most cases of acquired and transmitted drug resistance, the currently available first-line ART regimens containing 3TC and/or EFV or NVP should be urgently amended, or promptly switched to the second-line regimens	–	-Drug Resistance was rapidly rising in China in recent years (typically since 2012), and this rise was mainly driven by NNRTI resistance

Antiretroviral therapy (ART), combination antiretroviral therapy (cART), Not reported (NR), people with HIV (PWH), Protease inhibitors (PIs), ritonavir-boosted protease inhibitors (PI/r), Non-nucleoside reverse transcriptase inhibitor (NNRTIs), Nucleoside reverse transcriptase inhibitor (NRTIs), Integrase strand transfer inhibitors (INSTIs), C–C motif chemokine receptor (CCR5), perinatally HIV-infected (PHIV), prevention of mother-to-child transmission (PMTCT), pretreatment human immunodeficiency virus drug resistance (PDR), Dolutegravir (DTG), Efavirenz (EFV), Nevirapine (NVP), raltegravir (RAL), elvitegravir (EVG), bictegravir (BIC), emtricitabine (FTC), tenofovir alafenamide (TAF), lamivudine (3TC), abacavir (ABC), tenofovir disoproxil fumarate (TDF), Rilvipirine (RPV), integrase inhibitors (INIs), Ritonavir-boosted lopinavir (LPV/r), Zidovudine (ZDV), allele-specific polymerase chain reaction (AS-PCR), drug-resistant minority variants (DRMVs), Fostemsavir (FTR), Ibalizumab (IBA), Leronlimab (PRO 140), Islatravir (ISL), Lenacapavir (LEN)

A wide variety of countries were represented in the studies, with China (n = 5), South Africa (n = 4), UK (n = 4), USA (n = 3), Canada (n = 3), Ethiopia (n = 3), Brazil (n = 3), Switzerland (n = 3), Spain (n = 3), and Italy (n = 2) being the countries with the most involvement. Additionally, India (n = 2), Australia, Denmark, Lebanon, Romania, and Nigeria each contributed one article to the review.

Regarding the classes of ART, the non-nucleoside reverse transcriptase inhibitors (NNRTIs), nucleoside reverse transcriptase inhibitors (NRTIs), and protease inhibitors (PIs) were the most commonly studied drugs in relation to drug resistance and determinants for virologic failure. Review of included studies demonstrated that poor medication adherence, high HIV viral load, low CD4 level at the baseline and during therapy, co-infection presence, and advanced clinical stage of infection were the most prevalent factors associated with drug resistance and virological failure. Notably, drug resistance related to NNRTIs was more frequently reported than resistance to other types of ART, based on the reviewed studies.

## Discussion

The advent of Antiretroviral Therapy (ART) has revolutionized the management of HIV, transforming it from a fatal disease to a chronic condition. The availability and efficacy of ART have played a crucial role in suppressing HIV and improving the quality of life for millions of people living with HIV [70, 71]. However, despite the significant progress, a subset of individuals living with HIV still face virological failure, which remains a global public health challenge.

In this umbrella review, we sought to address virological failure and its risk factors by evaluating evidence from multiple studies. Our findings have shed light on several important factors that contribute to virological failure in people living with HIV. Poor adherence to treatment emerged as the most commonly reported contributing factor, as evidenced by multiple studies [31, 39, 42–45, 59, 63, 67]. This was followed by a lower CD4 count at the initiation of ART [31, 33, 42, 44, 45, 61]. Other reported factors include high viral load at the initiation of ART [33, 39, 45, 52, 61, 67], and the presence of co-infections [31]. Additionally, we noticed that several factors can lead to drug resistance in people living with HIV such as the long duration of ART.

Poor adherence to treatment can be caused by a variety of factors. Adherence to ART is crucial for its effectiveness, and factors such as difficulty following regimens, side effects of therapy, lack of health knowledge, and limited availability of ART due to formulary restrictions or costs were identified as barriers to adherence [72]. Moreover, stigma associated with HIV, substance use, low income, and the number of administered drugs were also associated with adherence issues [36, 46, 73, 74]. Several approaches have been suggested to enhance adherence to ART in people living with HIV. Agyeman-Yeboah J et al. suggested that education and counseling, adherence tools, health service delivery, and antiretroviral strategies could improve ART adherence [75]. Recognizing substance users in HIV primary care and developing, evaluating, and implementing integrated substance use-mental health-adherence therapies may also be clinically relevant goals for optimal disease management and secondary HIV prevention initiatives [74]. Additionally, various personal-level psychological interventions have been shown to improve ART adherence [76]. Treatment techniques, such as fixed-dose combinations of ART drugs to reduce dosing complexity, and educational activities, including pharmaceutical treatment management initiatives, have been shown to improve adherence to HIV therapy [72].

In individuals living with HIV, failure of antiretroviral therapy (ART) can result in increased medication toxicity and the development of drug resistance. Studies have shown that nearly half of those who fail first-line ART are at a higher risk of failing second-line treatment as well [77, 78]. Therefore, it is crucial to detect treatment failure at an early stage to prevent the emergence of further drug resistance. Plasma viral load monitoring is considered the gold standard for evaluating the success of ART [79]. In addition, lower CD4 cell count, an important factor in HIV replication, is another indicator of virological failure [80]. Immunocompromised patients with lower CD4 cell counts are at a higher risk of opportunistic infections, leading to increased viral replication and a higher risk of drug resistance [81, 82]. A study by Zoufaly A et al. showed that 12% of children with ART resistance had a CD4 cell count below 200 cells/μl [83]. Therefore, it is crucial to monitor CD4 cell count along with viral load regularly in people living with HIV to ensure effective treatment and prevent virological failure.

Additionally, the duration of ART use has implications for treatment success and drug resistance. Longer duration of ART use was associated with loss of information, poor adherence, and increased drug interruption, leading to virological failure [31, 33, 84–86]. In a study conducted by Zoufaly A et al., 53% of the children who experienced virological failure were on ART for an average of 3.5 years [83]. In contrast, in another study by Chen RY et al., the median duration of ART and successful ART regimens in 405 ART-naive individuals reported to be 1.6 years, indicating that successful treatment outcomes can be achieved with shorter durations of ART use, highlighting the importance of optimizing treatment regimens [87]. The occurrence of TB/HIV co-infection increases the likelihood of patients experiencing virological failure [88]. The consequences of TB/HIV co-infection are mutual, with TB accelerating the progression of HIV infection to AIDS, resulting in an increased risk of virological failure and death [89]. Additionally, the combination of TB and HIV infections lowers the CD4 cell count, leading to a weakened immune system [86].

Drug resistance is an inevitable concern in the treatment of HIV due to the virus's genetic diversity. However, using new strategies to produce medications may reduce the occurrence of drug resistance. Since the discovery of ARTs as a viable therapeutic medication, tremendous progress has been achieved, resulting in the FDA’s approval of five integrase strand transfer inhibitors (INSTIs): Dolutegravir (DTG), Raltegravir (RAL), Elvitegravir (EVG), Bictegravir (BIC), and Cabotegravir (CAB). INSTIs have been demonstrated to successfully block HIV-1 replication and have a more robust genetic threshold to resistance than nucleoside reverse transcriptase inhibitors (NRTIs) and non-nucleoside reverse transcriptase inhibitors (NNRTIs). Extensive studies have provided a thorough understanding of the resistance patterns of DTG in recent years. It has been demonstrated that DTG exhibits a prolonged binding half-life to HIV integrase (IN) compared to both RAL and EVG. This characteristic likely contributes to DTG's ability to remain effective against the majority of first-generation INSTI-resistant variants [90–93]. More intriguingly CAB is the first long-acting drug in HIV-1 therapy [94]. In this umbrella review, NNRTIs demonstrated the highest resistance and INSTIs the lowest. NNRTIs have exhibited more risk of resistance than NRTIs and DTG has a low risk of resistance [43, 53, 59]. DTG and lamivudine (3TC) can lower the viral load even in triple to dual regimen changes [56, 66, 69].

It is worth noting that regional differences play a role in the occurrence of ART resistance and virological failure. Sub-Saharan Africa, for example, faces a high prevalence of HIV treatment failure, partly due to factors such as inadequate nutritional assistance, delayed HIV testing, and treatment failure [86, 95–97] [98–100]. In China, resistance to ART has increased since 2012, primarily driven by NNRTI resistance [101].

## Conclusion

In conclusion, resistance to HIV drugs and virologic failure is a significant issue that affects the quality of life and well-being of HIV-infected individuals. This article has highlighted several primary causes of treatment failure, including poor adherence to medication, low CD4 T-cell counts, high viral levels, and specific types of regimens used. Interventions such as drug use, educational counseling, psychological support, and changes in management can help address these causes. With proper adherence to ART regimens, virologic failure without mutation is rare. However, considerations such as drug interactions must be taken into account and can be managed using online tools. Notably, Sub-Saharan Africa remains a region with high rates of virological failure, mainly due to delayed testing and diagnosis and the delayed initiation of ART. These challenges emphasize the urgent need for improved strategies to detect resistance in HIV patients and reduce the likelihood of viral failure by addressing its underlying causes. Further research is needed to improve the detection of resistance in HIV patients and reduce the likelihood of viral failure by identifying and addressing its underlying causes.

### Supplementary Information

**Additional file 1.** The search results of PubMed, Embase, Scopus, and Web of Science.

## Data Availability

The authors stated that all information provided in this article could be shared.

## References

[1] WHO. HIV 2021. https://www.who.int/data/gho/data/themes/hiv-aids#:~:text=Globally%2C%2038.4%20million%20%5B33.9%E2%80%93,considerably%20between%20countries%20and%20regions.

[2] Collaboration H-C, Ray M, Logan R, Sterne JA, Hernandez-Diaz S, Robins JM (2010). The effect of combined antiretroviral therapy on the overall mortality of HIV-infected individuals. AIDS.

[3] Safdari R, Ahmadi M, Bahaadinbeigy K, Farzi J, Noori T, Mehraeen E (2019). Identifying and validating requirements of telemental health services for Iranian veterans. J Family Med Primary Care.

[4] Mehraeen E, Safdari R, SeyedAlinaghi S, Noori T, Kahouei M, Soltani-Kermanshahi M (2020). A mobile-based self-management application- usability evaluation from the perspective of HIV-positive people. Health Policy Technol.

[5] Mehraeen E, Safdari R, Seyedalinaghi SA, Mohammadzadeh N, Arji G (2018). Identifying and validating requirements of a mobile-based self-management system for people living with HIV. Stud Health Technol Inform.

[6] Baeten JM, Kahle E, Lingappa JR, Coombs RW, Delany-Moretlwe S, Nakku-Joloba E (2011). Genital HIV-1 RNA predicts risk of heterosexual HIV-1 transmission. Sci Transl Med.

[7] Graham SM, Holte SE, Peshu NM, Richardson BA, Panteleeff DD, Jaoko WG (2007). Initiation of antiretroviral therapy leads to a rapid decline in cervical and vaginal HIV-1 shedding. AIDS.

[8] Vernazza PL, Troiani L, Flepp MJ, Cone RW, Schock J, Roth F (2000). Potent antiretroviral treatment of HIV-infection results in suppression of the seminal shedding of HIV. Swiss HIV Cohort Study AIDS.

[9] https://www.unaids.org/en/resources/fact-sheet. UGHAsFsAf.

[10] UNAIDS F-TT, UNAIDS, Editor. 2014.

[11] Severe P, Juste MA, Ambroise A, Eliacin L, Marchand C, Apollon S (2010). Early versus standard antiretroviral therapy for HIV-infected adults in Haiti. N Engl J Med.

[12] Mirzapour P, Motlagh F, SeyedAlinaghi S, Mehraeen E (2021). Comparison of the effectiveness of positive thinking training and acceptance and commitment therapy on quality of life and resilience of people living with HIV. HIV AIDS Rev Int J HIV Related Probl.

[13] Mehraeen E, Salehi MA, Behnezhad F, Moghaddam HR, SeyedAlinaghi S (2021). Transmission modes of COVID-19: a systematic review. Infect Disord Drug Targets.

[14] CDC. HIV in the United States. 2019.

[15] WHO. Consolidated guidelines on the use of antiretroviral drugs for treating and preventing HIV infection: recommendations for a public health approach, 2nd ed2016. https://www.who.int/publications/i/item/9789241549684.

[16] Shahmohamadi E, SeyedAlinaghi S, Karimi A, Behnezhad F, Mehraeen E, Dadras O (2021). HIV/HTLV-1 co-infection: a systematic review of current evidence. HIV & AIDS Rev Int J HIV Relat Probl.

[17] Castley A, Sawleshwarkar S, Varma R, Herring B, Thapa K, Dwyer D (2017). A national study of the molecular epidemiology of HIV-1 in Australia 2005–2012. PLoS ONE.

[18] Arimide DA, Abebe A, Kebede Y, Adugna F, Tilahun T, Kassa D (2018). HIV-genetic diversity and drug resistance transmission clusters in Gondar, Northern Ethiopia, 2003–2013. PLoS ONE.

[19] Maldarelli F, Kearney M, Palmer S, Stephens R, Mican J, Polis MA (2013). HIV populations are large and accumulate high genetic diversity in a nonlinear fashion. J Virol.

[20] Lessells R, Katzenstein D, De Oliveira T (2012). Are subtype differences important in HIV drug resistance?. Curr Opin Virol.

[21] Kiepiela P, Manasa J, Moosa M-Y, Moodley P, Gordon M, Parikh UM (2014). HIV drug resistance patterns at the epicentre of the HIV-1 epidemic in Kwazulu-Natal, South Africa 2003–2013. J AIDS Clin Res.

[22] Skhosana L, Steegen K, Bronze M, Lukhwareni A, Letsoalo E, Papathanasopoulos MA (2015). High prevalence of the K65R mutation in HIV-1 subtype C infected patients failing tenofovir-based first-line regimens in South Africa. PLoS ONE.

[23] Palmer NB, Salcedo J, Miller AL, Winiarski M, Arno P (2003). Psychiatric and social barriers to HIV medication adherence in a triply diagnosed methadone population. AIDS Patient Care STDS.

[24] McCluskey SM, Siedner MJ, Marconi VC (2019). Management of virologic failure and HIV drug resistance. Infect Dis Clin North Am.

[25] Parienti JJ, Bangsberg DR, Verdon R, Gardner EM (2009). Better adherence with once-daily antiretroviral regimens: a meta-analysis. Clin Infect Dis.

[26] NIH. ART Therapy in Adults and Adolescents. 2018. https://indd.adobe.com/view/0380886a-52e0-4434-80be-0da162be7d87.

[27] Nettles RE, Kieffer TL, Kwon P, Monie D, Han Y, Parsons T (2005). Intermittent HIV-1 viremia (Blips) and drug resistance in patients receiving HAART. JAMA.

[28] Sungkanuparph S, Overton ET, Seyfried W, Groger RK, Fraser VJ, Powderly WG (2005). Intermittent episodes of detectable HIV viremia in patients receiving nonnucleoside reverse-transcriptase inhibitor-based or protease inhibitor-based highly active antiretroviral therapy regimens are equivalent in incidence and prognosis. Clin Infect Dis.

[29] Hermans LE, Moorhouse M, Carmona S, Grobbee DE, Hofstra LM, Richman DD (2018). Effect of HIV-1 low-level viraemia during antiretroviral therapy on treatment outcomes in WHO-guided South African treatment programmes: a multicentre cohort study. Lancet Infect Dis.

[30] Aberg JA, Mills A, Moreno S, Slater J, Prakash M, Clark A (2023). The evolution of clinical study design in heavily treatment-experienced persons with HIV: a critical review. Antivir Ther.

[31] Agegnehu CD, Techane MA, Mersha AT, Atalell KA (2022). Burden and associated factors of virological failure among people living with HIV in Sub-Saharan Africa: a systematic review and meta-analysis. AIDS Behav.

[32] Almeida-Brasil CC, Moodie EEM, Cardoso TS, Nascimento ED, Ceccato M (2019). Comparison of the predictive performance of adherence measures for virologic failure detection in people living with HIV: a systematic review and pairwise meta-analysis. AIDS Care.

[33] Anderson K, Muloiwa R, Davies MA (2020). Long-term outcomes in perinatally HIV-infected adolescents and young adults on antiretroviral therapy: a review of South African and global literature. Afr J AIDS Res.

[34] Bernab Crossed D, Sign© KJ, Siedner M, Tsai AC, Marconi VC, Murphy RA (2022). Detection of HIV Virologic failure and switch to second-line therapy: a systematic review and meta-analysis of data from Sub-Saharan Africa. Open Forum Infect Dis.

[35] Bertagnolio S, Hermans L, Jordan MR, Avila-Rios S, Iwuji C, Derache A (2021). Clinical impact of pretreatment human immunodeficiency virus drug resistance in people initiating nonnucleoside reverse transcriptase inhibitor-containing antiretroviral therapy: a systematic review and meta-analysis. J Infect Dis.

[36] Bezabhe WM, Chalmers L, Bereznicki LR, Peterson GM (2016). Adherence to Antiretroviral therapy and virologic failure: a meta-analysis. Medicine (Baltimore).

[37] Blanco JL, Marcelin AG, Katlama C, Martinez E (2018). Dolutegravir resistance mutations: lessons from monotherapy studies. Curr Opin Infect Dis.

[38] Borges ÁH, Lundh A, Tendal B, Bartlett JA, Clumeck N, Costagliola D (2016). Nonnucleoside reverse-transcriptase inhibitor- vs ritonavir-boosted protease inhibitor-based regimens for initial treatment of HIV infection: a systematic review and meta analysis of randomized trials. Clin Infect Dis.

[39] Cevik M, Orkin C, Sax PE (2020). Emergent resistance to dolutegravir among instinaive patients on first-line or second-line antiretroviral therapy: a review of published cases. Open Forum Infect Dis.

[40] Cruciani M, Parisi SG (2019). Dolutegravir based antiretroviral therapy compared to other combined antiretroviral regimens for the treatment of HIV-infected naive patients: a systematic review and meta-analysis. PLoS ONE.

[41] Cruz ML, Cardoso CA (2015). Perinatally infected adolescents living with human immunodeficiency virus (Perinatally human immunodeficiency virus). World J Virol.

[42] Daltro ACB, Almeida CS, Unfried AGC, de Aquino TR, Travassos A (2023). Virological failure and adherence to antiretroviral therapy in adolescents and young adults living with human immunodeficiency virus. Trop Med Int Health.

[43] de Waal R, Lessells R, Hauser A, Kouyos R, Davies MA, Egger M (2018). HIV drug resistance in sub-Saharan Africa: public health questions and the potential role of real-world data and mathematical modelling. J Virus Erad.

[44] Diallo M, Adekpedjou R, Ahouada C, Ngangue P, Ly BA (2020). Impact of pre-antiretroviral therapy cd4 counts on drug resistance and treatment failure: a systematic review. AIDS Rev.

[45] Edessa D, Sisay M, Asefa F (2019). Second-line HIV treatment failure in sub-Saharan Africa: a systematic review and meta-analysis. PLOS ONE.

[46] Feng Q, Zhou AS, Zou HC, Ingle S, May MT, Cai WP (2019). Quadruple versus triple combination antiretroviral therapies for treatment naive people with HIV: systematic review and meta-analysis of randomised controlled trials. BMJ British Med J.

[47] Grau S, Miró JM, Olalla J, Alcalá JC, Castro A, Rubio-Rodríguez D (2023). Comparison of the design and methodology of Phase 3 clinical trials of bictegravir/emtricitabine/tenofovir alafenamide (BIC/FTC/TAF) and dolutegravir-based dual therapy (DTG) in HIV: a systematic review of the literature. Expert Rev Anti Infect Ther.

[48] Guo C, Wu Y, Zhang Y, Liu X, Li A, Gao M (2021). Transmitted drug resistance in antiretroviral therapy-naive persons with acute/early/primary HIV infection: a systematic review and meta-analysis. Front Pharmacol..

[49] Gupta RK, Gregson J, Parkin N, Haile-Selassie H, Tanuri A, Andrade Forero L (2018). HIV-1 drug resistance before initiation or re-initiation of first-line antiretroviral therapy in low-income and middle-income countries: a systematic review and meta-regression analysis. Lancet Infect Dis.

[50] Hauser A, Goldstein F, Reichmuth ML, Kouyos RD, Wandeler G, Egger M (2022). Acquired HIV drug resistance mutations on first-line antiretroviral therapy in Southern Africa: systematic review and Bayesian evidence synthesis. J Clin Epidemiol.

[51] Huang Y, Huang X, Luo Y, Zhou Y, Tao X, Chen H (2018). Assessing the efficacy of lopinavir/ritonavir-based preferred and alternative second-line regimens in HIV-infected patients: a meta-analysis of key evidence to support WHO recommendations. Front Pharmacol.

[52] Kabbara WK, Ramadan WH (2015). Emtricitabine/rilpivirine/tenofovir disoproxil fumarate for the treatment of HIV-1 infection in adults. J Infect Public Health.

[53] Kandel CE, Walmsley SL (2015). Dolutegravir—a review of the pharmacology, efficacy, and safety in the treatment of HIV. Drug Design Develop Ther.

[54] Karade S, Chaturbhuj DN, Sen S, Joshi RK, Kulkarni SS, Shankar S (2018). HIV drug resistance following a decade of the free antiretroviral therapy programme in India: a review. Int J Infect Dis.

[55] Kefale AT, Dadi TL, Biru TT, Mega TA (2018). Treatment outcome and adverse events of tenofovir disoproxil fumarate based regimens as compared to zidovudine based regimens among people living with HIV/AIDS: a systematic review and meta-analysis of observational studies. Open AIDS J.

[56] Llibre JM, Álvarez H, Yzusqui M (2018). Clinical impact of virological failure and resistance analysis definitions used in pivotal clinical trials of initial antiretroviral treatment: a systematic review. AIDS Rev.

[57] Mbuagbaw L, Mursleen S, Irlam JH, Spaulding AB, Rutherford GW, Siegfried N (2016). Efavirenz or nevirapine in three-drug combination therapy with two nucleoside or nucleotide-reverse transcriptase inhibitors for initial treatment of HIV infection in antiretroviral-naïve individuals. Cochrane Database Systematic Rev.

[58] Mbunkah HA, Bertagnolio S, Hamers RL, Hunt G, Inzaule S, Rinke De Wit TF (2020). Low-abundance drug-resistant HIV-1 variants in antiretroviral drug-naive individuals: a systematic review of detection methods, prevalence, and clinical impact. J Infect Dis.

[59] McCluskey SM, Pepperrell T, Hill A, Venter WDF, Gupta RK, Siedner MJ (2021). Adherence, resistance, and viral suppression on dolutegravir in sub-Saharan Africa: implications for the TLD era. AIDS.

[60] Muheem A, Baboota S, Ali J (2021). An in-depth analysis of novel combinatorial drug therapy via nanocarriers against HIV/AIDS infection and their clinical perspectives: a systematic review. Expert Opin Drug Deliv.

[61] Nozza S, Svicher V, Saracino A, d'Ettorre G, De Luca A, Maggiolo F (2015). State of the art of dual therapy in 2015. AIDS Rev.

[62] Patel R, Evitt L, Mariolis I, Di Giambenedetto S, d’Arminio Monforte A, Casado J (2021). HIV treatment with the two-drug regimen dolutegravir plus lamivudine in real-world clinical practice: a systematic literature review. Infect Dis Ther.

[63] Ssemwanga D, Lihana RW, Ugoji C, Abimiku A, Nkengasong J, Dakum P (2015). Update on HIV-1 acquired and transmitted drug resistance in Africa. AIDS Rev.

[64] Stella-Ascariz N, Arribas JR, Paredes R, Li JZ (2017). The role of HIV-1 drug-resistant minority variants in treatment failure. J Infect Dis.

[65] Temereanca A, Ruta S (2023). Strategies to overcome HIV drug resistance-current and future perspectives. Front Microbiol.

[66] Vannappagari V, Ragone L, Henegar C, van Wyk J, Brown D, Demarest J (2019). Prevalence of pretreatment and acquired HIV-1 mutations associated with resistance to lamivudine or rilpivirine: a systematic review. Antivir Ther.

[67] Wallis CL, Godfrey C, Fitzgibbon JE, Mellors JW (2017). Key factors influencing the emergence of human immunodeficiency virus drug resistance in low- and middle-income countries. J Infect Dis.

[68] Xing H, Ruan Y, Liao L, Shao Y (2022). The prevalence and related effects of HIV drug-resistant strains against antiretroviral therapy in China. Infect Microbes Dis.

[69] Zuo L, Liu K, Liu H, Hu Y, Zhang Z, Qin J (2020). Trend of HIV-1 drug resistance in china: a systematic review and meta-analysis of data accumulated over 17 years (2001–2017). EClinicalMedicine.

[70] Alinaghi SAS, Rasoolinejad M, Najafi Z, Dadras O, Malekianzadeh E, Mirzazadeh A (2019). Drug resistance patterns in HIV patients with virologic failure in Iran. Archiv Clin Infect Dis.

[71] Rasoolinejad M, Sarraf M, Najafi Z, SeyedAlinaghi S, Badie BM, Salehi M (2019). Virologic failure in different antiretroviral regimens among pediatric patients with HIV referring to a voluntary counseling and testing (VCT) Center in Tehran, Iran (2004–2017). Archiv Pediatric Infect Dis.

[72] Schaecher KL (2013). The importance of treatment adherence in HIV. Am J Manag Care.

[73] Katz IT, Ryu AE, Onuegbu AG, Psaros C, Weiser SD, Bangsberg DR (2013). Impact of HIV-related stigma on treatment adherence: systematic review and meta-synthesis. J Int AIDS Soc.

[74] Gonzalez A, Barinas J, O’Cleirigh C (2011). Substance use: impact on adherence and HIV medical treatment. Curr HIV/AIDS Rep.

[75] Agyeman-Yeboah J, Ricks EJ, Williams M, Jordan PJ, Ten Ham-Baloyi W (2022). Integrative literature review of evidence-based guidelines on antiretroviral therapy adherence among adult persons living with HIV. J Adv Nurs.

[76] Whiteley LB, Olsen EM, Haubrick KK, Odoom E, Tarantino N, Brown LK (2021). A review of interventions to enhance HIV medication adherence. Curr HIV/AIDS Rep.

[77] Enderis BO, Hebo SH, Debir MK, Sidamo NB, Shimber MS (2019). Predictors of time to first line antiretroviral treatment failure among adult patients living with HIV in public health facilities of Arba Minch Town. Southern Ethiopia Ethiop J Health Sci.

[78] Niemeyer K, King A, Mengistu S, Hennig N (2016). Predictors of antiretroviral therapy failure in an urban HIV/AIDS clinic in Addis Ababa. Ethiopia Lancet Global Health.

[79] Rutherford GW, Anglemyer A, Easterbrook PJ, Horvath T, Vitoria M, Penazzato M (2014). Predicting treatment failure in adults and children on antiretroviral therapy: a systematic review of the performance characteristics of the 2010 WHO immunologic and clinical criteria for virologic failure. AIDS.

[80] Okoye AA, Picker LJ (2013). CD 4+ T-cell depletion in HIV infection: mechanisms of immunological failure. Immunol Rev.

[81] Palladino C, Briz V, Bellón JM, Bartolo IS, Carvalho P, Camacho R (2013). Predictors of attrition and immunological failure in HIV-1 patients on highly active antiretroviral therapy from different healthcare settings in Mozambique. PLoS ONE.

[82] Adolescent-ARV-guide lines/0/. 2019. IAGftUoAAiH-IAaAAfaiNggha-a. Accessed 27 Aug 27 2012.

[83] Zoufaly A, Fillekes Q, Hammerl R, Nassimi N, Jochum J, Drexler JF (2013). Prevalence and determinants of virological failure in HIV-infected children on antiretroviral therapy in rural cameroon: a cross-sectional study. Antivir Ther.

[84] Boukli N, Boyd A, Collot M, Meynard J-L, Girard P-M, Morand-Joubert L (2018). Utility of HIV-1 DNA genotype in determining antiretroviral resistance in patients with low or undetectable HIV RNA viral loads. J Antimicrob Chemother.

[85] Clutter DS, Jordan MR, Bertagnolio S, Shafer RW (2016). HIV-1 drug resistance and resistance testing. Infect Genet Evol.

[86] Agegnehu CD, Techane MA, Mersha AT, Atalell KA (2022). Burden and associated factors of virological failure among people living with HIV in Sub-Saharan Africa: a systematic review and meta-analysis. AIDS Behav.

[87] Chen RY, Westfall AO, Mugavero MJ, Cloud GA, Raper JL, Chatham AG (2003). Duration of highly active antiretroviral therapy regimens. Clin Infect Dis.

[88] Getaneh T, Negesse A, Dessie G, Desta M (2022). The impact of tuberculosis co-infection on virological failure among adults living with HIV in Ethiopia: a systematic review and meta-analysis. J Clin Tuberc Other Mycobact Dis.

[89] WHOTHCRCMU. World Health Organization. TB/HIV coinfection regional clinical manual update. 2017.

[90] Quashie PK, Mesplède T, Wainberg MA (2013). Evolution of HIV integrase resistance mutations. Curr Opin Infect Dis.

[91] Hightower KE, Wang R, DeAnda F, Johns BA, Weaver K, Shen Y (2011). Dolutegravir (S/GSK1349572) exhibits significantly slower dissociation than raltegravir and elvitegravir from wild-type and integrase inhibitor-resistant HIV-1 integrase-DNA complexes. Antimicrob Agents Chemother.

[92] Métifiot M, Marchand C, Pommier Y (2013). HIV integrase inhibitors: 20-year landmark and challenges. Adv Pharmacol.

[93] Thierry E, Deprez E, Delelis O (2017). Different pathways leading to integrase inhibitors resistance. Front Microbiol.

[94] Mbhele N, Chimukangara B, Gordon M (2021). HIV-1 integrase strand transfer inhibitors: a review of current drugs, recent advances and drug resistance. Int J Antimicrob Agents.

[95] Gebremichael DY, Hadush KT, Kebede EM, Zegeye RT (2018). Food insecurity, nutritional status, and factors associated with malnutrition among people living with HIV/AIDS attending antiretroviral therapy at public health facilities in West Shewa Zone Central Ethiopia. BioMed Res Int.

[96] World Health Organization (2015). Consolidated guidelines on HIV testing services: 5Cs: consent, confidentiality, counselling, correct results and connection.

[97] Deribew A, Biadgilign S, Berhanu D, Defar A, Deribe K, Tekle E (2018). Capacity of health facilities for diagnosis and treatment of HIV/AIDS in Ethiopia. BMC Health Serv Res.

[98] Nash D, Tymejczyk O, Gadisa T, Kulkarni SG, Hoffman S, Yigzaw M (2016). Factors associated with initiation of antiretroviral therapy in the advanced stages of HIV infection in six Ethiopian HIV clinics, 2012 to 2013. J Int AIDS Soc.

[99] Ogoina D, Finomo F, Harry T, Inatimi O, Ebuenyi I, Tariladei W-w (2015). Factors associated with timing of initiation of antiretroviral therapy among HIV-1 infected adults in the Niger Delta Region of Nigeria. PloS ONE.

[100] Amare T, Getinet W, Shumet S, Asrat B (2018). Prevalence and associated factors of depression among PLHIV in Ethiopia: systematic review and meta-analysis, 2017. AIDS Res Treatment.

[101] Zuo L, Liu K, Liu H, Hu Y, Zhang Z, Qin J (2020). Trend of HIV-1 drug resistance in China: a systematic review and meta-analysis of data accumulated over 17 years (2001–2017). EClinicalMedicine..

