# Supplementary material for: Current ART, determinants for virologic failure and implications for HIV drug resistance: an umbrella review
Source: AIDS Res Ther. 2023 Oct 27;20:74. doi: 10.1186/s12981-023-00572-6 (PMC10604802; doi:10.1186/s12981-023-00572-6)
Supplement: Supplementary file 1 — Additional file 1. The search results of PubMed, Embase, Scopus, and Web of Science. [file 12981_2023_572_MOESM1_ESM.docx]

Pubmed Search Query

Time of search: 2023-05-26

Results: 241

((“HIV”[mesh] OR “Human Immunodeficiency Virus”[Title/Abstract] OR “HIV”[Title/Abstract] OR “AIDS Virus*”[Title/Abstract] OR “Acquired Immune Deficiency Syndrome Virus”[Title/Abstract] OR “Acquired Immunodeficiency Syndrome Virus”[Title/Abstract] OR “aids associated virus”[Title/Abstract] OR “aids related virus”[Title/Abstract] OR “immunodeficiency associated virus”[Title/Abstract]) AND (“Antiretroviral Therapy, Highly Active”[mesh] OR “Antiretroviral Therapy, Highly Active”[Title/Abstract] OR “anti-retroviral therapy”[Title/Abstract] OR “ART (drug therapy)”[Title/Abstract] OR “antiretroviral therapy”[Title/Abstract] OR “HAART”[Title/Abstract]) AND (“Drug Resistance, Viral”[mesh] OR “Drug Resistance, Viral”[Title/Abstract] OR “Antiviral Drug Resistance”[Title/Abstract] OR “Drug Resistance”[Title/Abstract] OR “antiviral resistance”[Title/Abstract] OR “virologic failure”[Title/Abstract]))

Embase Search Query

Time of search: 2023-05-26
Results: 341

(' Human immunodeficiency virus'/exp OR ‘Human Immunodeficiency Virus’:ab,ti OR ‘HIV’:ab,ti OR ‘AIDS Virus*’:ab,ti OR ‘Acquired Immune Deficiency Syndrome Virus’:ab,ti OR ‘Acquired Immunodeficiency Syndrome Virus’:ab,ti OR ‘aids associated virus’:ab,ti OR ‘aids related virus’:ab,ti OR ‘immunodeficiency associated virus’:ab,ti) AND (‘antiretroviral therapy’/exp OR ‘Antiretroviral Therapy, Highly Active’:ab,ti OR ‘anti-retroviral therapy’:ab,ti OR ‘ART (drug therapy)’:ab,ti OR ‘antiretroviral therapy’:ab,ti OR ‘HAART’:ab,ti) AND (‘drug resistance’/exp OR ‘antiviral resistance’/exp OR ‘Drug Resistance, Viral’:ab,ti OR ‘Antiviral Drug Resistance’:ab,ti OR ‘Drug Resistance’:ab,ti OR ‘antiviral resistance’:ab,ti OR ‘virologic failure’:ab,ti)

Scopus Search Query

Time of search: 2023-05-26
Results: 166

( TITLE-ABS (“Human Immunodeficiency Virus”) OR TITLE-ABS (“HIV”) OR TITLE-ABS ( “AIDS Virus*”) OR TITLE-ABS ( “Acquired Immune Deficiency Syndrome Virus”) OR TITLE-ABS ( “Acquired Immunodeficiency Syndrome Virus”) OR TITLE-ABS ( “aids associated virus”) OR TITLE-ABS ( “aids related virus”) OR TITLE-ABS ( “immunodeficiency associated virus”)) AND (TITLE-ABS (“Antiretroviral Therapy, Highly Active”) OR TITLE-ABS (“anti-retroviral therapy”) OR TITLE-ABS ( “ART (drug therapy)”) OR TITLE-ABS ( “antiretroviral therapy”) OR TITLE-ABS ( “HAART”)) AND ( TITLE-ABS (“Drug Resistance, Viral”) OR TITLE-ABS(“Drug Resistance, Viral”) OR TITLE-ABS ( “Antiviral Drug Resistance”) OR TITLE-ABS ( “Drug Resistance”) OR TITLE-ABS ( “antiviral resistance”) OR TITLE-ABS ( “virologic failure”))

Web of science Search Query

Time of search: 2023-05-26
Results: 281

(TS=(“ Human Immunodeficiency Virus”) OR TS=(“HIV”) OR TS=(“AIDS Virus*”) OR TS=(“Acquired Immune Deficiency Syndrome Virus”) OR TS=(“Acquired Immunodeficiency Syndrome Virus”) OR TS=(“aids associated virus”) OR TS=(“aids related virus”) OR TS=(“immunodeficiency associated virus”)) AND (TS=(“ Antiretroviral Therapy, Highly Active”) OR TS=(“anti-retroviral therapy”) OR TS=(“ART (drug therapy)”) OR TS=(“antiretroviral therapy”) OR TS=(“HAART”)) AND (TS=(“ Drug Resistance, Viral”) OR TS=(”Drug Resistance, Viral”) OR TS=(“Antiviral Drug Resistance”) OR TS=(“Drug Resistance”) OR TS=(“antiviral resistance”) OR TS=(“virologic failure”))
